# Imaging Diagnosis—Intestinal Leiomyositis in a 3‐Year‐Old Labrador Retriever

**DOI:** 10.1111/vru.70093

**Published:** 2025-10-02

**Authors:** Mercedes Cantu, Eric T. Hostnik, Rebecca Urion

**Affiliations:** ^1^ Department of Veterinary Clinical Sciences, College of Veterinary Medicine The Ohio State University Columbus Ohio USA

## Abstract

A 3‐year‐old male neutered Labrador Retriever was originally presented to his primary care veterinarian for a 3–4‐week history of hyporexia, a 1‐week history of anorexia, and vomiting. There was moderate gastrointestinal (GI) distention with heterogeneous soft tissue luminal material within the small intestine in screening abdominal radiographs. Referral and computed tomography were performed, identifying a severe generalized dilation of the GI tract but no obstructive lesion. Severe functional intestinal ileus without obstruction was diagnosed. The patient underwent laparotomy with full‐thickness intestinal surgical biopsies due to presumptive infiltrative enteropathy, yielding a diagnosis of intestinal leiomyositis.

## 1 Signalment, History, and Clinical Findings

A 3‐year‐old male neutered Labrador Retriever was originally presented to his primary care veterinarian for a 3–4‐week history of hyporexia followed by 1 week of anorexia and vomiting. The patient had demonstrated historical dietary indiscretion, so a mechanical obstruction was presumed. The primary care veterinarian performed abdominal radiographs and an abdominocentesis. The peritoneal fluid was a flocculant yellow/brown color with evidence of debris and rod‐shaped bacteria that were too numerous to count. The patient was then referred to The Ohio State University emergency service for the suspicion of bowel perforation and sepsis. Point‐of‐care ultrasound examination revealed markedly distended small intestinal segments containing echogenic fluid and scant anechoic peritoneal effusion. Blood work was performed, which showed a mild normocytic (67 fL; rr 62–77 fL), hyperchromic (38.1 g/dL; rr 33–36.1 g/dL), nonregenerative anemia (Hct 34%; rr 40%–59%), and mild hypokalemia (3.56 mmol/L; rr 4.1–5.4 mEq/L).

## 2 Imaging, Diagnosis, and Outcome

**FIGURE 1:**
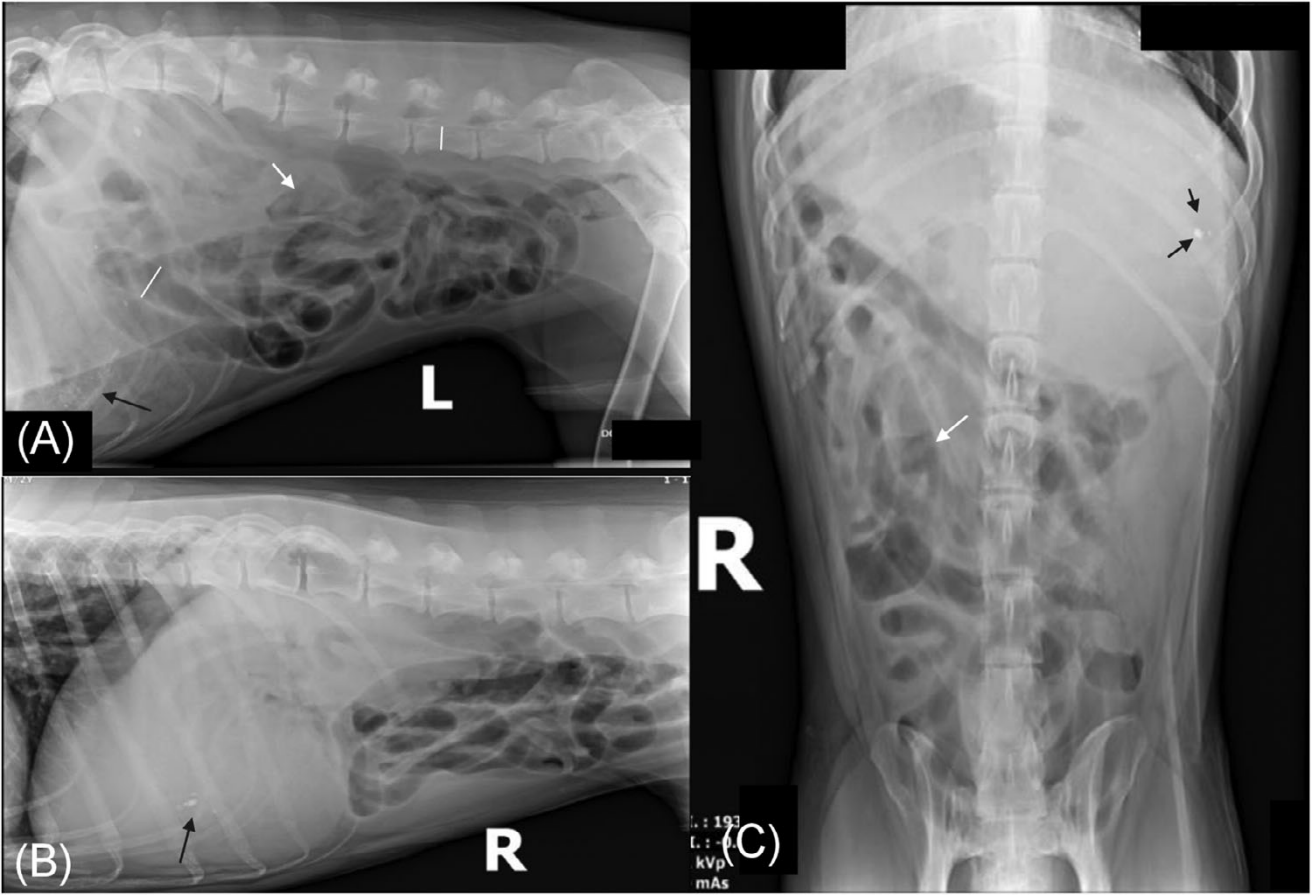
Abdominal radiographs: (A) left lateral, (B) right lateral, and (C) ventrodorsal. (A) The small intestine is diffusely, moderately gas‐distended. The small intestinal segments measure less than 2× the height of L5 (white lines). (A and C) There is scant heterogeneous luminal material within the small intestine (white arrows). (A–C) The stomach is distended with homogeneous soft tissue and several small, ovoid mineral opacities (black arrows). The serosal detail is mildly decreased.

Abdominal radiographs performed by the referring veterinarian revealed poor serosal detail and a diffusely gas‐distended gastrointestinal (GI) tract (Figure 1). The stomach also contains a moderate amount of homogeneous soft tissue and multiple small, ovoid mineral opacities (Figure 1). The intestinal segments were moderately, diffusely dilated with no evidence of segmental distention. The bowel segments measured less than 2× the height of L5 (Figure 1A). Scant heterogeneous soft tissue luminal content was within a few small intestinal segments (Figure 1A,C).

Thoracic radiographs were performed for routine screening. There was no pulmonary metastasis, intrathoracic lymphadenopathy, or aspiration pneumonia, but the stomach remained distended with homogeneous soft tissue and gas.

A triple‐phase contrast‐enhanced abdominal computed tomography (CT) angiography study was performed under moderate sedation in sternal recumbency using a 64‐detector scanner (GE Revolution EVO, GE Healthcare). Images were acquired in the transverse plane using the following image acquisition parameters: helical scan mode, 120 kVp, fluctuating mA with a max of 250, slice thickness = 1.25 mm, pitch = 1. Positive contrast medium was administered intravenously (2 mL/kg of Omnipaque 240 mgI/mL Iohexol injection, Amersham Health Inc.).

**FIGURE 2:**
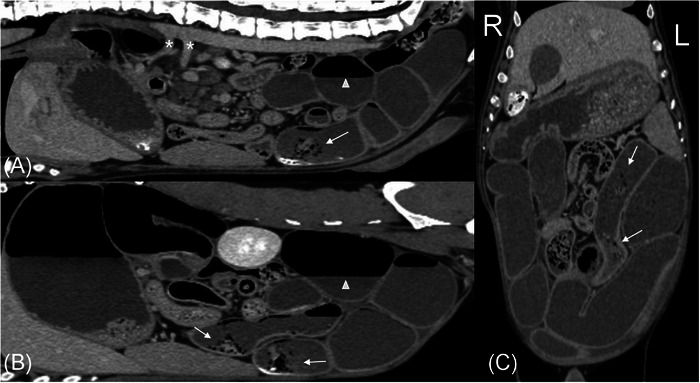
Post‐contrast venous phase CT images in mid‐sagittal (A), left parasagittal (B), and dorsal (C) planes with the patient in sternal recumbency. Cranial is to the left in (A) and (B) and to the top in (C). The majority of the small intestine is moderately diffusely dilated with a mixture of homogeneous soft tissue and gas, resulting in gravity‐dependent gas–fluid interfaces (white arrowheads). A small amount of heterogeneous soft tissue, mineral, and gas attenuating content is within several small intestinal segments, although incompletely filling the lumens (white arrows). In (A), there is normal contrast‐filling of the celiac and cranial mesenteric arteries (white asterisks).

**FIGURE 3:**
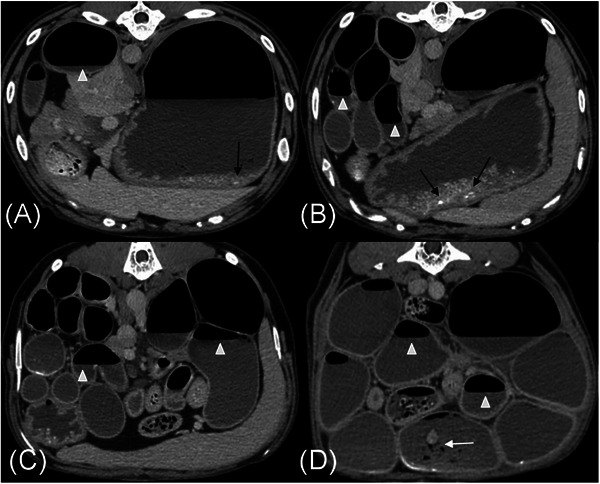
Transverse post‐contrast venous phase CT images with the patient in sternal recumbency, with panels A–D arranged from cranial to caudal. The patient's left is on the right side of the images. There is moderate gastric and small intestinal dilation in all images. The gastric granular mineral identified radiographically persists (black arrows). Stratification of homogeneous soft tissue and gas with gravity dependent gas–fluid interfaces (white arrowheads) are throughout most small intestinal segments. Small collections of granular mineral and heterogeneous soft tissue are within several small intestinal segments but incompletely filling the lumens (white arrow). Gastric and small intestinal mural thickness and contrast‐enhancement are normal.

Abdominal CT revealed a moderately distended stomach with gas, heterogeneous soft tissue, and granular mineral, and severely, diffusely dilated small intestine (approximately 4× the height of L5) that was filled with fluid, gas, and variable amounts of granular mineral and heterogeneous soft tissue. There was a stratification of the fluid and gas within the small bowel, resulting in horizontal gas–fluid interfaces suggestive of reduced to absent peristalsis (Figures 2A,B, and 3). The granular mineral and heterogeneous soft tissue within the small bowel did not occupy the entire lumen and were not deemed obstructive (Figures 2 and 3), resulting in an imaging diagnosis of diffuse functional ileus. The GI wall thickness was uniform, with no areas of abnormal mural thickening or altered contrast enhancement. Normal vascular enhancement was identified in all phases of the CT angiogram, excluding macroscopic thromboembolic disease or mesenteric torsion/volvulus as a cause for generalized severe ileus (Figure 2A). No peritoneal pathology was identified. As all other differential diagnoses were excluded, an underlying diffuse enteropathy was prioritized.

An exploratory laparotomy was performed, which confirmed a lack of mechanical obstruction. Following gastric decompression, minimal peristalsis was observed. The small intestine was diffusely, severely dilated, thin‐walled, and erythematous with multifocal petechiation. No peristaltic waves were observed in the majority of the small intestine, but minimal movement was observed in the ileum. Full‐thickness biopsies were obtained of the stomach, duodenum, jejunum, ileum, and a jejunal lymph node. The biopsies of the stomach, duodenum, jejunum, and ileum revealed a mild to moderate, diffuse, subacute to chronic, lymphoplasmacytic gastroenteritis and leiomyositis with multifocal neutrophilic infiltrates, and smooth muscle vacuolation. Gastric luminal spiral bacteria (*Helicobacter* spp.) and yeast (*Cyniclomyces guttulatus*) were also identified. According to the pathologist, the pathogenic potential of these organisms was uncertain, as these can be found in unaffected or clinically normal animals but have also been associated with gastritis in dogs.

Following surgery, the patient was medically managed with supportive care. Due to a lack of therapeutic response, euthanasia was pursued 3 days later.

## 3 Discussion

Intestinal pseudo‐obstruction (or functional ileus) is an uncommon clinical syndrome in human medicine, with even fewer cases reported in veterinary medicine. This syndrome is characterized by impaired intestinal motility without evidence of obstruction of the gut lumen [1]. In human medicine, pseudo‐obstructive syndromes are either acute (postoperative ileus, peritonitis, hypokalemia, and myocardial infarction) or, more commonly, chronic in nature [2, 3]. Intestinal pseudo‐obstruction can further be classified as secondary to other pathological conditions (developmental, infectious, inflammatory, autoimmune, metabolic, paraneoplastic, endocrine, and toxic etiologies) or idiopathic [4]. Chronic intestinal pseudo‐obstruction has been reported in dogs, horses, cats, and birds [4, 5, 6, 7]. Interestingly, the patient in this case demonstrated hypokalemia, but its role in the clinical presentation is uncertain given the relatively mild nature.

Leiomyositis is a disease that affects the ability of the enteric smooth muscle to contract appropriately due to the infiltration of lymphocytes between functional myocytes, which in turn results in ileus [4]. Canine patients present with clinical signs that are nonspecific, including abdominal pain, nausea, vomiting, regurgitation, bloating, diarrhea, anorexia, abdominal distension, and weight loss [8]. The pathogenesis of intestinal leiomyositis in dogs is not known, but in humans, there have been reports of it developing secondary to a primary autoimmune disease [9], secondary to gastroenteritis, and to congenital enteric neuromuscular disease [10], with the most common form being idiopathic [4]. Overall, this syndrome has been poorly documented in dogs, with only sporadic case reports or limited case series. Full‐thickness biopsies of the GI tract are required for diagnosis because the lesions typically involve the muscularis layer [1], ideally performed at multiple sites throughout the GI tract. The overall prognosis of dogs with pseudo‐obstruction secondary to intestinal leiomyositis is poor, with most of the dogs being euthanized shortly after diagnosis [4].

Treatment of chronic intestinal pseudo‐obstruction in human medicine is variable, with most treatments aimed at improving quality of life. Treatments include enteral nutrition, total/home parenteral nutrition, drugs aimed at GI propulsive activity, immunosuppressive treatments, antibiotics for bacterial overgrowth due to GI stasis, and surgical therapy [2, 11]. In veterinary medicine, treatments have included combined antiemetic, prokinetic, antimicrobial, and immunomodulatory agents [8].

Based on a literature review, there is one prior publication with a brief written description of the CT findings for a case of intestinal leiomyositis in dogs [4], in addition to a few reports of abdominal radiographs and ultrasound findings. This manuscript contributes a visual record of intestinal leiomyositis in a dog. Radiographic findings generally demonstrate severe gastric distention and diffuse small intestinal dilation [4, 8]. Abdominal ultrasound findings include poor to absent peristalsis and diffusely dilated small intestine. There have also been reports of loss of gastric and jejunal wall layering, hypoechoic liver, hypoechoic pancreas, hyperechoic mesentery, and mesenteric lymphadenopathy [4, 8]. In the prior report of CT findings of a patient with confirmed leiomyositis, the scan revealed segmentally to diffusely dilated small intestine as well as gastric distension with no evidence of mechanical obstruction [4], similar to the current case.

In this case, a CT scan was performed for multiple reasons. Due to the patient's size, ultrasound would have been challenging and likely would have provided an incomplete evaluation of the stomach and intestine due to the degree of luminal gas. Additionally, the significant weight loss over such a short period and initial consideration of peritoneal sepsis were concerning for a possible aggressive process, necessitating a comprehensive examination of the entire abdomen. Abdominal ultrasonography would have provided a greater assessment of GI wall thickness and layering, but given the original concern for mechanical obstruction, a more complete evaluation of the GI lumen was considered to be of greater importance. The CT findings in this case were consistent with other cases of leiomyositis evaluated radiographically and ultrasonographically [4, 8], and CT effectively excluded mechanical obstruction, wall thickness alteration, macroscopic vascular disease, or peritoneal disease as underlying causes. Identifying peritoneal adhesions is difficult, even on CT, but the bowel contour was not considered compatible with adhesions in this case. A cause for the initial peritoneocentesis results was not ultimately confirmed. Although a small pocket of encapsulated peritoneal fluid may have been under‐recognized on CT, the authors speculate that the bowel lumen may have been sampled inadvertently, as a source of bowel leakage was not identified surgically.

This report highlights the imaging features of intestinal leiomyositis in a canine patient, as well as the utility of CT angiography in cases of GI ileus, particularly for larger patients in which other imaging modalities may be limited. CT excluded mechanical causes of ileus and gross peritoneal or vascular disease, and although definitive diagnosis for this condition still requires surgical biopsies, the use of CT helped guide diagnostic and therapeutic decision‐making.

## Ethics Statement

This study was conducted in accordance with the ethical standards outlined by the institutional animal care and use guidelines. All procedures involving animals were performed as part of necessary diagnostic or therapeutic work‐up and were not conducted for the purpose of research alone. No experimental procedures were performed. The welfare of the animals was a primary consideration throughout, and all efforts were made to minimize stress and discomfort. The authors affirm that this work complies with the principles of veterinary medical ethics.

## Consent

Informed client consent was obtained prior to all diagnostic imaging and medical interventions.

## Conflicts of Interest

Dr. Eric T. Hostnik is Editor‐in‐Chief of Veterinary Radiology & Ultrasound. Dr. Hostnik recused himself from any decision about manuscript acceptance to this journal. Authors were anonymized for the review process. The authors declare no other conflicts of interest.
